# Memory Impairment in Relapsing-Remitting Multiple Sclerosis Using a Challenging Semantic Interference Task

**DOI:** 10.3389/fneur.2020.00309

**Published:** 2020-04-21

**Authors:** Jordi A. Matias-Guiu, Ana Cortés-Martínez, Rosie E. Curiel, Alfonso Delgado-Álvarez, Aníbal Fernández-Oliveira, Vanesa Pytel, Paloma Montero, Teresa Moreno-Ramos, David A. Loewenstein, Jorge Matías-Guiu

**Affiliations:** ^1^Department of Neurology, Hospital Clinico San Carlos, Health Research Institute “San Carlos” (IdISCC), Universidad Complutense de Madrid, Madrid, Spain; ^2^Department of Psychiatry and Behavioral Sciences, Center for Cognitive Neuroscience and Aging, Miller School of Medicine, University of Miami, Miami, FL, United States; ^3^Department of Neurology, Neurological Institute, Oviedo, Spain

**Keywords:** memory, multiple sclerosis, semantic, proactive interference, neuropsychological assessment

## Abstract

**Objective:** Episodic memory is frequently impaired in Multiple Sclerosis (MS), but the cognitive characteristics and neuropsychological processes involved remain controversial. Our aim was to study episodic memory dysfunction in MS, using the LASSI-L, a novel memory-based cognitive stress test that uses a new paradigm that capitalizes on semantic interference.

**Methods:** Cross-sectional study in which 93 patients with MS (relapsing-remitting) and 124 healthy controls were included. The LASSI-L test was administered to all participants, as well as a comprehensive neuropsychological battery including a selective reminding test. MS patients were divided into two groups, with cognitive impairment (CI-MS) and cognitively preserved (CP-MS).

**Results:** Reliability of the LASSI-L test was high (Cronbach's alpha 0.892) and there were less ceiling effects. MS patients scored lower than controls on all LASSI-L subtests, except for maximum storage of the initial target items (CRA2). Effect sizes were moderate-large. A delay in learning, difficulties in retroactive semantic interference, failure to recover from proactive semantic interference, and delayed recall were the most frequent findings in MS patients. Scores associated with maximum storage capacity, and retroactive semantic interference were the most strongly associated with cognitive impairment and employment status.

**Conclusion:** We found that deficits in maximum learning, difficulties in recovery from the effects of proactive semantic interference and retroactive semantic interference are three important breakdowns in episodic memory deficits among patients with MS. The LASSI-L showed good psychometric and diagnostic properties. Overall, our study supports the utility of the LASSI-L, as a new cognitive test, useful for neuropsychological assessment in MS in clinical and research settings.

## Introduction

Multiple sclerosis (MS) is a chronic, predominantly autoimmune-mediated disease of the central nervous system. Main events in its pathophysiology include inflammation, demyelination, axonal damage, and neurodegeneration (1). MS is the most common cause of non-traumatic neurological disability in young adults (2). MS has a wide breadth of clinical symptoms, and cognitive impairment is now recognized among the most disabling and common deficits. Previous studies have found prevalence rates of cognitive impairment ranging from 40 to 70% (3). The neuropsychological profile in MS is mainly characterized by deficits in attention and executive functioning, information processing speed, and episodic memory (4). These cognitive deficits negatively impact daily living activities, employment, and quality of life (5).

Episodic memory includes three main cognitive processes: encoding, storage, and retrieval. The neuropsychological processes underlying memory impairment in MS are debatable (6). Early research linked episodic memory deficits in MS to a selective retrieval failure (7), where encoding and storage abilities were preserved. Patients with MS show the tendency to improve with cued recall more so than when required to free recall, or with recognition tasks (8). In contrast, other mechanisms of memory, such as a reduced amount of information acquisition in initial trials has been described (9). Subsequent investigations have shown that patients with MS require more trials during learning to reach the same level of storage than a healthy population, but once information is consolidated, recall and recognition are comparable to healthy controls (10). Some investigators have argued that problematic mechanisms involving consolidation of information in MS span beyond mere deficits in attention or executive function (6).

Interference is one of the processes associated with forgetting of learned material, in which past or new memories interacts with the capacity to learn or remember new or past information, respectively [see Loewenstein et al. (11)]. With proactive interference, the recent learning of new information limits the ability to store new similar information. For instance, when learning the names of people one meets during a party hinders accurately learning additional names during the remainder of the party. Conversely, retroactive interference is the acquisition of new memories during consolidation that leads to forgetting the previously learned information. Using the previous example, retroactive interference is said to occur if learning the names of the additional people resulted in loss of the names acquired at the beginning of the party. Because such interference in memory is likely to occur in daily living, tests evaluating this phenomenon could potentially be considered more ecologically relevant. However, relatively few studies have assessed interference in MS. In this setting, some studies have found increased effects of retroactive interference in memory ability among MS patients while others have not found significant differences in comparison to healthy controls (7, 12–16). Regarding proactive interference, there are also controversial results in the previous literature (12, 14). First, they use heterogeneous memory tasks, some of them not specifically focused on producing a prominent proactive and/or retroactive interference effects. In this regard, not all studies examined semantic interference, or words did not share the same semantic category. And third, some studies include a relatively low sample size or used patients with heterogeneous memory performance.

In MS, studies regarding memory dysfunction have generally used the Selective Reminding Test (SRT), California Verbal Learning Test (CVLT), Rey Auditory Verbal Learning Test, the Wechsler Memory Scale, or other experimental tasks (15). Although many of these tests share some aspects, such as selective reminding or employing multiple learning trials, they also have some differences in the procedure of memory assessment. This issue, as well as the heterogeneity of MS itself, could probably explain the variable findings observed across different studies (6). Because memory deficits are frequent in MS and several neuropsychological processes are involved in its pathophysiology, it is important to employ instruments to properly examine the patients. Proper assessment therefore measures learning, response to cues, and adequately taps both proactive and retroactive interference. There is increasing evidence that semantic interference tasks may be more sensitive to detect subtle memory deficits and underlying brain dysfunction than traditional memory assessments (17).

The Loewenstein-Acevedo Scale for Semantic Interference and Learning (LASSI-L) is a novel test first developed with the aim to detect preclinical and prodromal stages of Alzheimer's disease (18). Several studies have been conducted in the setting of Alzheimer's disease, showing a high diagnostic accuracy and a correlation with amyloid load, limbic connectivity, and brain volumes involved during the first stages of Alzheimer's disease (19–22). Furthermore, the LASSI-L has shown to be superior to the Free and Cued Selective Reminding Test (FCSRT) in the prediction of Alzheimer's disease features on ^18^F-Fluorodeoxyglucose positron emission tomography imaging (23). Cognitive profiles of MS and Alzheimer's disease are distinct in that attention and processing speed are more impaired than episodic memory in MS, while memory dysfunction is predominant in early stages of Alzheimer's disease, but certain overlap has been found between MS and amnestic mild cognitive impairment (24). Interestingly, the LASSI-L captures several memory processes, including encoding, storage and learning, free and cued recall, delayed recall, proactive and retroactive interference and, uniquely, the ability to recover from the effects of proactive semantic interference (18). The LASSI-L is a challenging and more demanding task than traditional cognitive assessments (17). Thus, it has been considered as a “cognitive stress test,” allowing the detection of more subtle memory deficits than is possible with other tools. This test is based on the examination of the semantic vulnerability that occurs during learning in patients on the Alzheimer's disease continuum, which is present even earlier than the impairment of storage and learning. The LASSI-L scores examining the different memory processes can be compared and checked against each other, and thus, they could be interpreted independently from demographic factors, which may represent an advantage in the absence of normative data (22). In addition, the test elicits frequent semantic intrusion errors, which have shown to detect biomarkers including amyloid positivity in the context of amnestic mild cognitive impairment (17). Although the LASSI-L was developed and validated for the early detection of Alzheimer's disease, this paradigm could also be particularly important in the setting of MS, which impairs young people in working age with strong memory requirements in daily living. Thus, sensitive neuropsychological tools to detect subtle changes that could impair functionality are urgently required to properly assess this population. The LASSI-L and the CVLT seem to share some characteristics as list-learning tests that employ two lists, but there are several important distinctions between the two measures. For one, relative to the CVLT, the LASSI-L relies on several cognitive paradigms that build up during the course of the administration. First, the measure utilizes an active semantic encoding process that is structured from the outset to minimize individual differences in learning strategies that can help or hinder memory encoding. To achieve this, the LASSI-L explicitly tells the participant that they will be presented with words that belong to one of three semantic categories. In contrast, the CVLT, does not make the categories explicit at the outset, thus, the examinee is required to generate an individualized learning strategy to organize their encoding. Other important distinctions involve the use of category cues and a 1:1 ratio of semantically similar target words on Lists A and B on the LASSI-L. This measure uses category cues during both encoding and retrieval in an attempt to maximize initial learning of the first target List A. By maximizing learning of the target List A, this potentiates interference effects when the person is challenged to learn a second list of competing target words. These cues along with the 1:1 competing targets on the LASSI-L also serve to elicit considerably more proactive and retroactive semantic interference and semantic intrusion errors. These semantic deficits, and especially semantic intrusions have been associated with increased risk of neurodegeneration and frontal executive hypoactivation on fMRI in middle-aged offspring of patients with late-onset Alzheimer's disease, which may suggest deficits in source memory and disinhibition (19–21, 24–26). The CVLT does measure proactive interference because it has a group words on List B that semantically compete with List A. The LASSI-L has a unique feature, which is a score that measures the failure to recover from proactive semantic interference, which is not measured by the CVLT. Retroactive semantic interference is elicited on both measures, but more robustly on the LASSI-L as compared to the CVLT, again due to the LASSI-L's use of category cues to encode, retrieve and the 1:1 semantic sharing of categories for every item on the word lists.

We hypothesized that LASSI-L, as a challenging memory task, may be applicable in MS, and may show differences between healthy controls and those with MS, and among patients with MS, differentiate those with and without cognitive impairment. Thus, in this study, our aims were threefold: first, to examine the psychometric properties of LASSI-L in MS; second, to evaluate the characteristics of memory dysfunction in MS patients, taking advantage of the unique memory measurement paradigms that comprise the LASSI-L; and third, to analyze the discrimination between patients with MS who are cognitively impaired vs. cognitively unimpaired, and between MS patients and cognitively healthy controls using the LASSI-L.

## Methods

### Study Design and Participants

We conducted a cross-sectional study involving 93 patients with MS (relapsing-remitting) and 124 healthy controls [HC]. MS and HC groups did not show differences in age, sex, and education. Mean age of MS patients and HC was 45.14 ± 10.79 and 43.60 ± 10.29 years (*p* = 0.288). Sixty-five (69.9%) and 83 (66.9%) were women (*p* = 0.377), and years of education were 16.35 ± 5.71 and 15.28 ± 3.34 (*p* =0.084) in MS and HC groups, respectively. Median EDSS was 2.50 [1.0–4.5], and disease duration was 13.81 ± 8.72 years. Patients with MS met the 2010 revised McDonald criteria (27). All participants were native Spanish speakers and had no prior or current history of other neurological, neurodevelopmental, psychiatric, or systemic diseases beyond MS that could potentially cause cognitive dysfunction. Prior depression was permitted, because of the relationship with MS, but none of the patients met criteria for clinical depression according to DSM-V at the time of assessment (28). MS patients with recent relapses (<12 weeks) were also excluded. The LASSI-L test was administered to all participants in the study. Patients with MS were also examined using a comprehensive neuropsychological battery comprised of the following tests: forward and backward conditions of Digit Span, Corsi Block-tapping Test, parts A and B of the Trail Making Test (TMT), Symbol Digit Modalities Test (SDMT), Boston Naming Test (BNT), Judgment Line Orientation (JLO), Rey-Osterrieth Complex Figure (ROCF) (copy and recall at both 3 and 30 min), Free and Cued Selective Reminding Test (FCSRT), verbal fluencies (animals and words beginning with “p” in 1 min), Stroop Color Word Interference Test, Tower of London (ToL), and the 3-s version of the Paced Auditory Serial Addition Test [PASAT]. This battery was chosen because it includes measures that are commonly used in MS batteries (i.e., SDMT, JLO, a version of SRT, etc.), there is comprehensive normative data available in Spain (29, 30), and it has been previously applied in large clinical and neuroimaging studies in MS (31–33). We also assessed fatigue and depression using the Fatigue Severity Scale (FSS) (34) and the Beck Depression Inventory (BDI) (35), respectively. Neuropsychological examination was performed by a trained neuropsychologist.

Verbal episodic memory in this battery is measured mainly with the FCSRT. In this version, 16 items are presented as words on stimulus cards (four cards including for words per card) (36). The subject is asked to read aloud the words. Items are non-prototypical words belonging to 16 semantic categories (for instance, “*celery*” in vegetables). When the individual has read the entire card, the examiner asks the subject to identify the name according to the semantic category. After a non-semantic interference task lasting 20 s (e.g., counting down), free recall is performed during 90 s. Items not remembered spontaneously are then cued by the examiner. Three free and cued recall trials are conducted following the same procedure. If the subject does not remember the item after the cue, the examiner gives the correct response during the first and second learning trials. Finally, a delayed free and cued recall after 30 min is performed.

MS patients were divided into two groups, with cognitive impairment (CI-MS) and cognitively preserved (CP-MS), according to the neuropsychological battery. Patients were classified as having cognitive impairment when two or more cognitive domains displayed an age- and education-adjusted percentile ≤ 5 according to the criteria specified elsewhere (31). The LASSI-L was independently administered during a separated session (<2 weeks apart) from the rest of the neuropsychological battery to avoid interferences with other tasks, and it was not used for diagnosis and group classification.

### Standard Protocol Approval and Patient Consents

The study was conducted with the approval of our hospital's Ethics Committee and all participants gave written informed consent.

### LASSI-L

The Spanish version of the LASSI-L was administered according to the procedure previously described (18, 22). Briefly, a list (List A) of 15 common words (five fruits, five musical instruments, and five articles of clothing) is presented to the patient, who is asked to read them aloud. The subject has 4 s to read aloud each item. Then, the examinee is asked to recall the words, firstly free (Free Recall 1, FRA1) and, afterwards, when prompted with a semantic cue (Cued Recall 1, CRA1). List A is presented again using the same method, followed again by cued recall (Cued Recall 2, CRA2). A semantically related list (List B) with 15 common words (also five fruits, five musical instruments, and five articles of clothing) is presented using the same procedure. Free Recall 1 (FRB1), Cued Recall 1 (CRB1) and Cued Recall 2 (CRB2) are performed. Then, the participant is asked to freely recall the words and on a subsequent trial, recall the words when prompted with cues from List A (Short-delay free recall, SdFRA, and Short-delay cued recall, SdCRA). Finally, a delayed recall (DR) of both lists is performed 20 min later (Table 1). Number of words correctly remembered and intrusions were registered for each trial. Time for trials is 60 s for each free recall, 20 s for each semantic category in cued recalls, and 90 s for the DR. LASSI-L administration of the two lists takes 8 min, followed by a 20 min interval until delayed recall is performed. Overall, the time of administration is 30 min.

**Table 1:** Summary of LASSI-L scores.

**Score**	**Description**	**Main memory process involved**
FRA1	Free Recall List A Trial 1	Encoding
CRA1	Cued Recall List A Trial 1	Initial learning
CRA2	Cued Recall List B Trial 2	Maximum storage
FRB1	Free Recall List B Trial 1	Proactive semantic interference (free recall)
CRB1	Cued Recall List B Trial 1	Proactive semantic interference (cued recall)
CRB2	Cued Recall List B Trial 2	Recovery from proactive semantic interference
SdFRA	Short Delay Free Recall List A	Retroactive interference (free recall)
SdCRA	Short Delay Cued Recall List A	Retroactive interference (cued recall)
DR	Delayed Recall	Free Delayed Recall

Two types of intrusions were computed for each score: intrusions from other list (e.g., i-CRB2), and total intrusions (related or unrelated to the other list) (e.g., ti-CRB2). We calculated the number of total intrusion errors as well as the percentage of intrusion errors ratio, which was computed to CRB1 (indicative of proactive semantic interference), CRB2 (revealing the recovery from proactive semantic interference), and SdCRA (showing the retroactive semantic interference effects). The formula of the percentage of intrusion errors ratio (PIE) is as follows: total intrusion errors / (total intrusion errors + number of correct responses), for each score (37).

### Statistical Analysis

Statistical analysis was performed using SPSS Statistics 20.0. Descriptive data are shown as mean ± standard deviation or median [interquartile range]. Internal consistency was estimated with Cronbach's alpha. Pearson's coefficient (r) was used for the analysis of correlations between quantitative variables. Frequency graphs were performed to evaluate the presence of ceiling and floor effects.

Pearson's correlation coefficient was also used to evaluate the influence of demographic factors (age, years of education) on obtained LASSI-L scores. Chi-squared or T-student tests were used to compare between two groups, when appropriate. ANOVA analyses with *post hoc* Tukey test were used to evaluate differences between the groups HC, CP-MS, and CI-MS. Effect size was estimated with Cohen's *d* for two means comparison, considering the effect as small (*d* = 0.2), moderate (*d* = 0.5), or large (*d* = 0.8). Effect size for ANOVA tests was assessed with eta squared, considering the effect as small (eta squared = 0.010), moderate (0.058), or large (0.137). A *p* < 0.01 was considered statistically significant. The alpha level was set at 0.01, instead of the more usual 0.05, to reduce the chance of a false positive result because of multiple comparisons, as the LASSI-L has several scores.

In addition, we estimated Z-scores for each LASSI-L subscales according to the mean and standard deviation from the HC group. Scores were subsequently dichotomized as impaired or non-impaired considering a cutoff point of *z* = −1.0. Intersections between LASSI-L scores were represented using UpSetR (38).

## Results

### Psychometric Characteristics of the LASSI-L

Internal consistency measured by the Cronbach's alpha for the 9 scores was 0.892. In patients with MS, the correlation was 0.26 (*p* = 0.012) between FRA1 and FCSRT (free recall 1), 0.42 (*p* < 0.001) between CRA2 and FCSRT (total recall), and 0.52 (*p* < 0.001) between DR and FCSRT (free delayed recall). Thus, convergent validity with FCSRT scores was generally moderate. All LASSI-L scores showed low (*r* < 0.29) correlations with fatigue and depression scales. No significant correlation was observed with EDSS (*p* > 0.05). All correlations are displayed in Table 2 and Supplementary Material S1.

**Table 2:** Correlations between LASSI-L scores and FCSRT, FSS, BDI, and EDSS.

	**FRA1**	**CRA1**	**CRA2**	**FRB1**	**CRB1**	**CRB2**	**SdFRA**	**SdCRA**	**DR**
FCSRT 1-minute free recall	0.265^*^	0.265^*^	0.364^**^	0.295^**^	0.295^**^	0.346^**^	0.354^**^	0.347^**^	0.465^**^
FCSRT total free recall	0.347^**^	0.363^**^	0.503^**^	0.401^**^	0.343^**^	0.462^**^	0.479^**^	0.449^**^	0.581^**^
FCSRT total recall	0.255^*^	0.374^**^	0.527^**^	0.354^**^	0.304^**^	0.506^**^	0.422^**^	0.430^**^	0.570^**^
FCSRT delayed free recall	0.361^**^	0.360^**^	0.466^**^	0.384^**^	0.342^**^	0.505^**^	0.498^**^	0.465^**^	0.564^**^
FCSRT delayed total recall	0.346^**^	0.400^**^	0.597^**^	0.407^**^	0.303^**^	0.541^**^	0.388^**^	0.354^**^	0.524^**^
FSS	−0.265^*^	−0.228^*^	−0.179	−0.294^**^	−0.244^*^	−0.201	−0.191	−0.206^*^	−0.283^**^
BDI	−0.289^**^	−0.219^*^	−0.226^*^	−0.219^*^	−0.192	−0.284^**^	−0.126	−0.165	−0.267^**^
EDSS	−0.029	0.005	−0.103	−0.007	−0.138	0.003	−0.061	−0.049	0.012

FCSRT, Free and Cued Selective Reminding Test; FSS, Fatigue Severity Scale; BDI, Beck Depression Inventory; EDSS, Expanded Disability Status Scale.

* p < 0.05.

** *p < 0.01*.

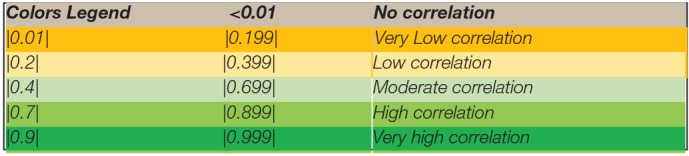

**Figure 1:**
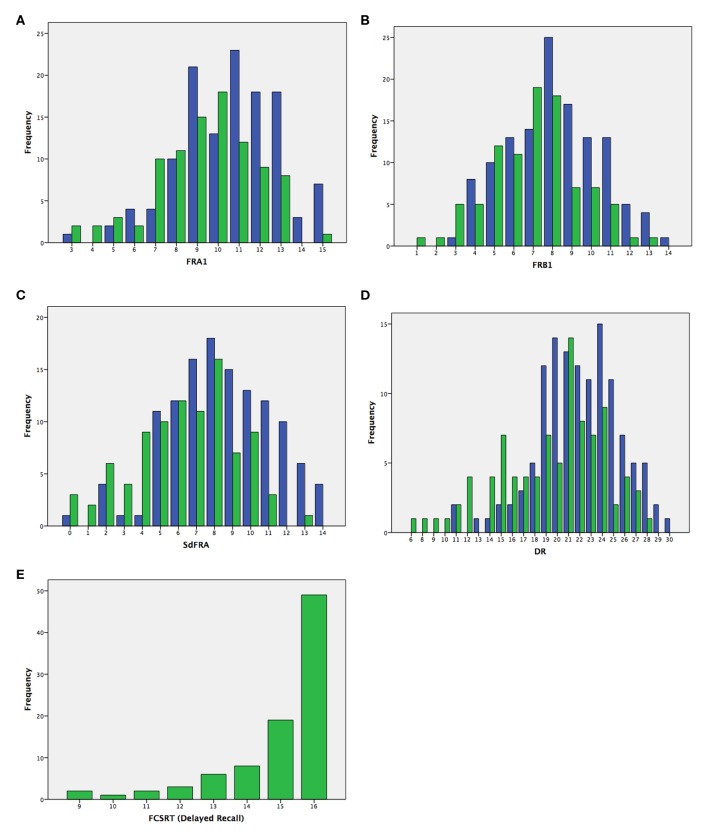
Frequency of scores in MS patients (green) and HC (blue) in the LASSI-L scores [**(A)** FRA1; **(B)** FRB1; **(C)** SdFRA; **(D)** DR] and in the Free and Cued Selective Reminding Test [**(E)** total delayed recall].

As shown in Figure 1, there was less ceiling and floor effects in LASSI-L (DR score), in contrast to FCSRT, in which a notable ceiling effect was observed.

### Test Performance in MS Patients in Comparison to HC

There were statistically significant differences on almost all LASSI-L measures between MS and HC groups (Table 3). Specifically, MS groups showed lower scores regarding FRA1, CRA1, FRB1, CRB1, CRB2, SdFRA, SdCRA, and DR (all *p* ≤ 0.001). The only score in which there were no statistically significant differences was CRA2 (maximum storage of list A) (*p* = 0.053). Effect sizes were moderate for FRA1 (*d* = 0.50), CRA1 (*d* = 0.62), CRB2 (*d* = 0.51), SdFRA (*d* = 0.75), SdCRA (*d* = 0.62), and DR (*d* = 0.60).

**Table 3:** Mean comparison between MS *vs*. HC on LASSI-L scores.

**Score and group**		**Mean ± SD**	***T*-test**	***p*-value**	**Cohen's *d***
FRA1	HC	10.60 ± 2.37	3.684	**<0.001**	0.50
	MS	9.39 ± 2.41			
CRA1	HC	11.07 ± 1.96	4.485	**<0.001**	0.62
	MS	9.73 ± 2.32			
CRA2	HC	13.46 ± 1.48	1.950	0.053	0.26
	MS	13.01 ± 1.90			
FRB1	HC	8.17 ± 2.39	3.505	**0.001**	0.48
	MS	7.03 ± 2.31			
CRB1	HC	8.89 ± 5.08	3.392	**0.001**	0.41
	MS	7.22 ± 2.51			
CRB2	HC	12.27 ± 1.91	3.781	**<0.001**	0.51
	MS	11.19 ± 2.29			
SdFRA	HC	8.46 ± 2.87	5.465	**<0.001**	0.75
	MS	6.32 ± 2.81			
SdCRA	HC	9.67 ± 2.65	4.534	**<0.001**	0.62
	MS	8.02 ± 2.65			
DR	HC	22.02 ± 3.64	4.474	**<0.001**	0.60
	MS	19.47 ± 4.72			

*Statistically significant p-values are shown in bold*.

**Figure 2:**
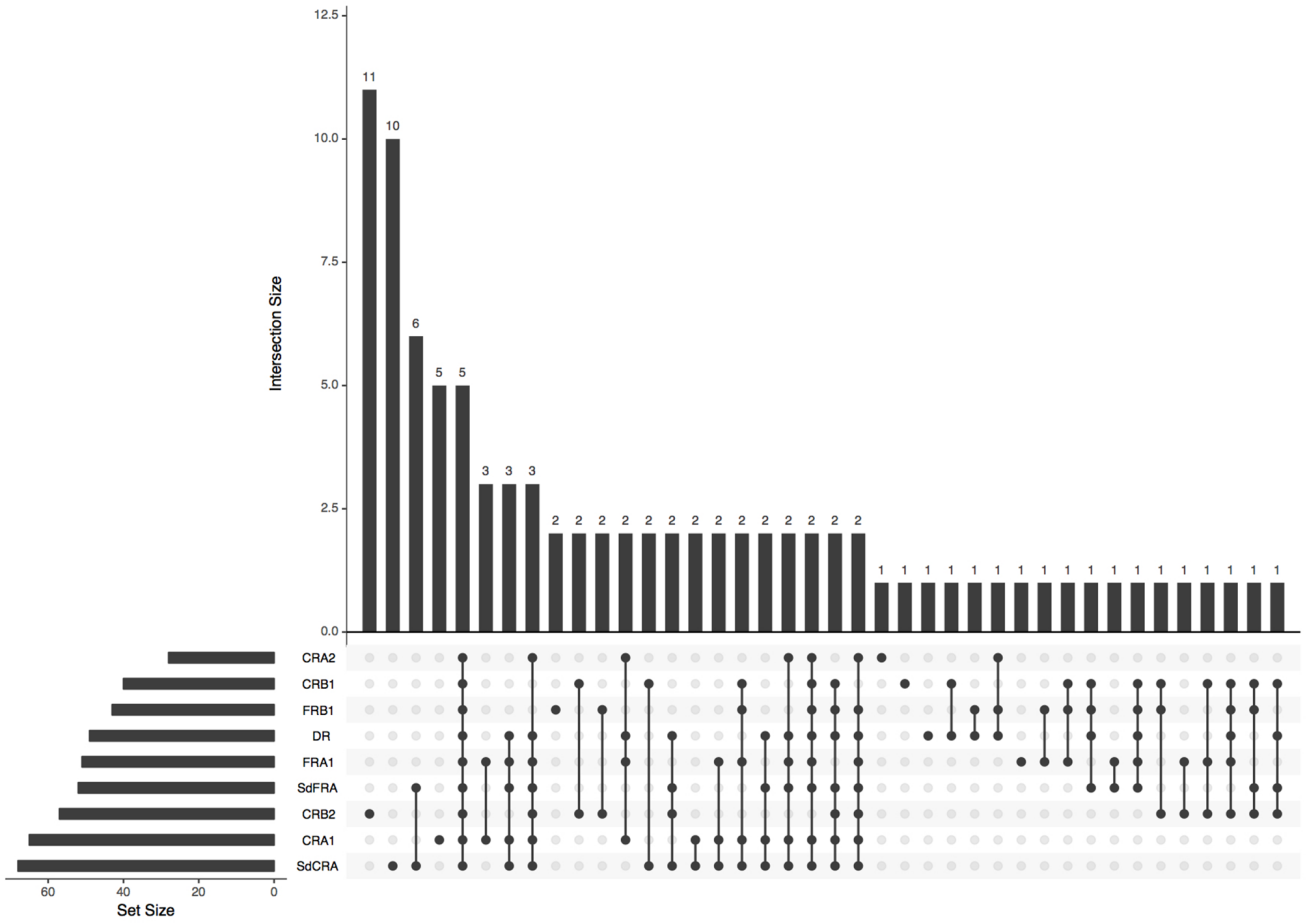
Plot of intersections between LASSI-L subscales (*z*-scores <-1.0) in MS patients. The bar chart on the left indicates the total number of patients showing LASSI-L score below the cutoff. The upper bar chart shows the number of cases displaying the intersection. Dark connected dots on the bottom panel indicate which subscales are considered for each intersection.

Using z-scores and considering a cutoff point of *z* = −1.0, the most frequent LASSI-L subscales impaired were SdCRA, CRA1, CRB2, and FRA1. Most frequent combinations of impairment were SdFRA-SdCRA, and the impairment of all the scores (Figure 2).

### Test Performance Across Groups HC, CP-MS, and CI-MS

There were no statistically significant differences in age [*F*_(2, 214)_ = 1.135, *p* = 0.323], years of education [*F*_(2, 214)_ = 1.754, *p* = 0.175], and sex (*X*^2^ 0.301, *p* = 0.860) between HC, CP-MS, and CI-MS. 41 patients (44.08%) were classified as CI-MS, while 52 (55.91%) were regarded as CP according to the previously specified criteria. When comparing demographic factors and disease characteristics in CP-MS and CI-MS, patients with cognitive impairment had longer disease duration (13.93 ± 8.43 vs. 13.71 ± 9.04, *p* < 0.001) and were older (46.12 ± 8.37 vs. 44.37 ± 12.40, *p* < 0.001), but there were no differences regarding sex (women were 68.3 vs. 71.2%, *p* = 0.860), years of education (16.73 ± 7.84 vs. 16.06 ± 3.20, *p* = 0.286) and EDSS (2.50 [1.0–5.5] vs. 2.25 [1.0–4.0], *p* = 0.532).

Table 4 shows the results for the LASSI-L scores in HC, CP-MS, and CI-MS. ANOVA test revealed a significant group effect for all LASSI-L scores (all *p* < 0.01). Effect sizes were large for CRA2 (η^2^ = 0.139), SdFRA (η^2^ = 0.165), SdCRA (η^2^ = 0.138), and DR (η^2^ = 0.153), and moderate for FRA1 (η^2^ = 0.065), CRA1 (η^2^ = 0.121), FRB1 (η^2^ = 0.074), and CRB2 (η^2^ = 0.090). *Post-hoc* analysis showed statistically significant differences between HC and CI-MS in FRA1, CRA1, CRA2, FRB1, CRB2, SdFRA, SdCRA, and DR, with HC showing greater results in all scores. In addition, lower scores were observed between in CI-MS when comparing with CP-MS in CRA2, CRB2, SdFRA, SdCRA, and DR. Conversely, no statistically significant differences were found between HC and CP-MS in CRA2 (maximum storage of list A).

**Table 4:** LASSI-L performance (scores) across diagnostic groups.

**Score**	**HC**	**CP-MS**	**CI-MS**	***F*_**(2, 214)**_**	***p*-value**	**Eta squared**
FRA1	10.60 ± 2.37^b^	9.63 ± 2.31	9.07 ± 2.52	7.42	**0.001**	0.065
CRA1	11.07 ± 1.96^b^	10.27 ± 2.23	9.05 ± 2.29	14.76	**<0.001**	0.121
CRA2	13.46 ± 1.48^b^	13.81 ± 1.22^c^	12.00 ± 2.13	17.25	**<0.001**	0.139
FRB1	8.17 ± 2.39^b^	7.50 ± 1.97	6.44 ± 2.59	8.59	**<0.001**	0.074
CRB1	8.48 ± 2.87	7.31 ± 2.49	7.10 ± 2.57	5.79	**0.004**	0.051
CRB2	12.27 ± 1.91^b^	11.67 ± 2.17^c^	10.59 ± 2.31	10.53	**<0.001**	0.090
SdFRA	8.46 ± 2.87^b^	7.17 ± 2.12^c^	5.24 ± 3.23	21.12	**<0.001**	0.165
SdCRA	9.67 ± 2.65^b^	8.87 ± 2.31^c^	6.95 ± 2.69	17.13	**<0.001**	0.138
DR	22.02 ± 3.64^b^	21.00 ± 3.50^c^	17.54 ± 5.36	19.37	**<0.001**	0.153

HC, healthy controls; CP-MS, Multiple Sclerosis cognitively preserved; CI-MS, Multiple Sclerosis cognitively impaired.

Statistically significant p-values are shown in bold (p < 0.01).

ANOVA with post-hoc analysis showed statistically significant differences between

a HC vs. CP-MS,

b HC vs. CI-MS, and

c *CP-MS vs. CI-MS*.

### Semantic Intrusion Errors Across Groups HC, CP-MS, and CI-MS

The MS group showed more intrusions in all scores in comparison to healthy controls (Supplementary Material S2). As depicted in Table 5, ANOVA showed a significant group effect for the number of total intrusions and intrusions from the other list in all the LASSI-L scores (all *p* < 0.01). Effect sizes were moderate for almost all total intrusions and intrusions from other list in scores susceptible to the effects of proactive interference, recovery from proactive interference, and retroactive interference.

**Table 5:** LASSI-L intrusions across diagnostic groups (total intrusions, intrusions from other list, and percentage of intrusion errors).

**Score**	**HC**	**CP-MS**	**CI-MS**	**F_**(2, 214)**_**	***p*-value**	**Eta squared**
ti-FRA1	0.10 ± 0.33^a^	0.54 ± 0.85	0.32 ± 0.65	11.30	**<0.001**	0.096
ti-CRA1	0.28 ± 0.64^a^^,^ ^b^	0.71 ± 0.93	0.73 ± 1.00	7.70	**0.001**	0.067
ti-CRA2	0.10 ± 0.35^b^	0.35 ± 0.59	0.44 ± 0.74	8.54	**<0.001**	0.074
ti-FRB1	0.40 ± 0.84^b^	0.75 ± 1.11	0.98 ± 0.12	6.20	**0.002**	0.055
i-FRB1	0.40 ± 0.78	0.85 ± 1.28	0.85 ± 0.98	5.88	**0.003**	0.052
ti-CRB1	1.10 ± 1.75^a^^,^ ^b^	2.23 ± 2.33	2.29 ± 2.14	9.15	**<0.001**	0.079
i-CRB1	1.01 ± 1.66^a^^,^ ^b^	2.13 ± 2.24	2.17 ± 2.07	9.68	**<0.001**	0.083
ti-CRB2	0.74 ± 1.26^a^^,^ ^b^	1.63 ± 1.91	1.63 ± 1.44	9.73	**<0.001**	0.083
i-CRB2	0.71 ± 1.12^a^^,^ ^b^	1.48 ± 1.77	1.51 ± 1.36	8.97	**<0.001**	0.077
ti-SdFRA	0.61 ± 1.08^b^	1.17 ± 1.45	1.71 ± 2.00	10.45	**<0.001**	0.089
i-SdFRA	0.56 ± 1.02^b^	1.06 ± 1.37	1.54 ± 1.96	8.91	**<0.001**	0.077
ti-SdCRA	1.11 ± 1.44^a^^,^ ^b^	1.96 ± 1.60	2.68 ± 2.57	13.80	**<0.001**	0.114
i-SdCRA	1.06 ± 1.42^b^	1.67 ± 1.51	2.41 ± 2.28	11.13	**<0.001**	0.094
PIE CRB1	0.10 ± 0.15^a^^,^ ^b^	0.20 ± 0.18	0.23 ± 0.20	11.38	**<0.001**	0.096
PIE CRB2	0.05 ± 0.08^a^^,^ ^b^	0.10 ± 0.11	0.12 ± 0.11	10.86	**<0.001**	0.092
PIE SdCRA	0.10 ± 0.13^a^^,^ ^b^	0.15 ± 0.14^c^	0.25 ± 0.19	15.46	**<0.001**	0.126

Statistically significant p-values are shown in bold (p < 0.01).

ANOVA with post-hoc analysis showed statistically significant differences between

a HC vs. CP-MS,

b HC vs. CI-MS, and

c CP-MS vs. CI-MS.

*ti-FRA1, total intrusions; i-FRA1, intrusions from other list; PIE, percentage of intrusions index*.

A significant group effect was also observed for the PIE index evaluating proactive interference effect (PIE CRB1) (*p* < 0.001), recovery from proactive interference (PIE CRB2) (*p* < 0.001), and retroactive interference (PIE SdCRA) (*p* < 0.001). Effect sizes were moderate for PIE-CRB1 (η^2^ = 0.096) and PIE-CRB2 (η^2^ = 0.092), and large for PIE-SdCRA (η^2^ = 0.126). *Post-hoc* analysis showed that CP-MS and CI-MS had a greater PIE in all the measures, in comparison to HC. CI-MS showed the most robust PIE ratio specifically in PIE-SdCRA.

### Comparison Between MS Patients With and Without Formal Disability

Twenty patients (21.5%) had a permanent recognized disability, while 73 (78.4%) were employed or had a recent employment. Patients with recognized disability were older and had longer times since the clinical onset, and scored lower on LASSI-L scores, as depicted in Table 6.

**Table 6:** Comparison between MS patients according to the recognition of formal disability.

**Score**	**Formal disability**	**Employment**	***T*-test**	***p*-value**
Age	51.10 ± 6.45	44.12 ± 10.24	3.717	**0.001**
Years of evolution of disease	18.80 ± 9.11	12.43 ± 8.15	3.014	**0.003**
Years of education	17.45 ± 10.97	16.05 ± 3.07	0.968	0.336
FRA1	7.75 ± 2.53	9.84 ± 2.19	−3.645	**<0.001**
CRA1	8.00 ± 2.02	10.21 ± 2.18	−4.239	**<0.001**
CRA2	11.20 ± 2.33	13.51 ± 1.42	−4.216	**<0.001**
FRB1	5.35 ± 2.23	7.49 ± 2.13	−3.940	**<0.001**
CRB1	5.80 ± 1.85	7.60 ± 2.54	−2.952	**0.004**
CRB2	9.75 ± 2.33	11.59 ± 2.12	−3.354	**0.001**
SdFRA	3.85 ± 2.73	7.00 ± 2.45	−4.958	**<0.001**
SdCRA	6.10 ± 2.57	8.55 ± 2.43	−3.813	**<0.001**
DR	15.20 ± 4.56	20.64 ± 4.07	−5.163	**<0.001**

*Statistically significant p-values are shown in bold (p < 0.01)*.

## Discussion

In this study, we found suitable psychometric properties for the utility of the LASSI-L in the setting of MS. Internal consistency was high, and we observed moderate correlations with a traditional verbal memory task that uses a common selective reminding paradigm. Conversely, correlations with fatigue or depression were low. Specifically, 22 of the 45 correlations of all LASSI-L and FCSRT scores were in the moderate range and 23 were low, and 42 of 45 were statistically significant considering a *p* < 0.01 as threshold. In some scores such as DR all five of five correlations were moderate with the FCSRT scores. In contrast, 13 of 22 correlations between depression and fatigue with LASSI-L were in the low range and the other five were very low correlations. In the correlation between LASSI and fatigue/depression scales, only five of 22 correlations were statistically significant. In addition, no significant correlation was found with EDSS, overall confirming that non-cognitive issues do not significantly impact LASSI-L performance. Tolerance of the LASSI-L in our sample was excellent and no patient failed to complete the test once it was started. Furthermore, there were less ceiling effects on this measure, in comparison to FCSRT in our cohort. Because MS usually affects young people of working age, it is important to have sensitive tools for cognitive assessment with low ceiling effects. Furthermore, almost all LASSI-L subscales showed statistically significant differences between MS patients and healthy controls. Several LASSI-L subscales also differentiated between cognitively preserved and cognitively impaired patients with MS.

Patients with MS displayed lower performance than controls in initial encoding (FRA1), similar to findings in prior literature (6). Moreover, they also demonstrated more difficulties in learning new information and were more vulnerable to the effects of proactive and retroactive semantic interference, with a lower ability to recover from the impact of proactive semantic interference, despite being given a second opportunity to learn the competing targets. While initial encoding and learning were impaired (FRA1, CRA1), MS patients reached a similar performance to HC during the second trial (CRA2). This is consistent with previous research suggesting difficulties in initial encoding and acquisition, which may be solved with successive trials (6). However, during the second list learning, CRB2, MS patients had lower scores, revealing difficulties in their ability to recover from the proactive interference effect. Notably, impaired performance on the CRB2 subscale was one of the most frequent deficits observed, confirming the failure to recovery from semantic proactive interference in MS patients (Figure 2). Similarly, mean LASSI-L scores associated with retroactive interference effects showed the largest effect sizes between patients and controls. Patients with MS also showed more intrusions, especially in the first trials during learning and in cued recall susceptible to retroactive interference. Overall, this suggests that, according to the assessment by LASSI-L, MS patients show a greater susceptibility to retroactive interference, failure to recovery from proactive interference, and a delay in the initial learning.

LASSI-L revealed a greater susceptibility to retroactive effects in MS patients displaying cognitive impairment. Conversely, there was no clear impact of proactive interference in CI-MS in comparison to the CP-MS group in the initial learning of the second list (CRB1); but CI-MS showed difficulties in improving their performance after the effect of proactive interference and, consequently, CRB2 scores were lower than CP-MS. Effect size for scores associated with retroactive interference were larger than for the other memory processes. Although our aim was not to compare MS with patients with Alzheimer's disease, LASSI-L showed a distinct memory dysfunction profile in MS with regards to the well-identified failure to recover from proactive interference, followed by deficits in maximum learning, as the greatest impairments in the earliest stages of Alzheimer's disease. On the other hand, the current investigation demonstrated a greater role of retroactive interference for MS relative to AD patients. This might reflect a combination of both episodic memory and executive dysfunction (4). Further, the LASSI could be useful in aged multiple sclerosis patients, in which the detection of comorbid Alzheimer's disease is challenging (24, 39). Specific studies with the LASSI-L in the older patients with MS are necessary to confirm these findings.

Intrusions were more frequent in the MS group than in healthy controls. Semantic intrusions are designed to be readily triggered on the LASSI-L because of the administration of two semantically related learning lists, and they are one of the primary challenges associated with the test. Difficulties in monitoring previously encoded information and in retrieval may underlie the production of intrusions (17). Furthermore, in some cases, the patients deliberately produced semantic intrusions using a strategy similar to the generation of a categorical verbal fluency in order to mask their absence of recall of the list. Because there are probably many mechanisms underlying semantic intrusions, the variability across individuals is greater and the effect sizes for intrusions were lower than for the other scores. Likewise, no significant differences were found in *post-hoc* analysis between CI-MS and CP-MS when considering the raw number of intrusion errors; however, when percentage of error intrusions ratios were taken into account, statistically significant differences were found between CI-MS and CP-MS in retroactive interference (PIE SdCRA). On the one hand, it suggests that the analysis of the intrusions is more useful in the context of the other scores. On the other hand, it supports the important role of retroactive interference in memory dysfunction in MS patients.

Our study employed a large number of well-characterized MS and healthy control subjects and employed a novel cognitive stress test that elicited strong proactive and retroactive interference effects among MS participants. The comparison between MS patients and healthy controls is often used in the literature for reporting new cognitive scales in MS. In addition, a cognitively impaired vs. cognitively unimpaired group of MS participants could be distinguished based upon LASSI-L measures as well as employed vs. disabled MS groups. We added this comparison in three groups (healthy controls, MS cognitively preserved, and MS with cognitive impairment) to investigate potential differences in memory dysfunction according to the cognitive status of MS patients. The observation of statistically significant differences in LASSI-L between patients showing cognitive impairment and those cognitively preserved also supports the validity of the test. Further, the LASSI-L was not used in initial diagnostic formulations and groupings, which is a tautological issue in a number of studies in which variables used to confer diagnosis are then validated as independent predictors of these same diagnoses.

Our study has some limitations that should be considered. We used a cross-sectional design and only relapsing-remitting MS patients were included. Thus, our results could not be generalized to progressive forms of the disease. Furthermore, because we strictly followed the norms of administration of LASSI-L, a recognition task is absent, which limits the assessment of retrieval deficits in delayed recall. We considered a *z* = −1 cutoff point to evaluate those scores of the LASSI-L which were below the mean in MS patients. Although there is no definite consensus about the cutoffs, *z* = −1 may be considered rather liberal. However, in some studies this cutoff resulted in an adequate balance between sensitivity and specificity (40). Future studies providing normative data in young populations for LASSI-L may be useful in the setting of MS in order to better define the impairment or not of LASSI-L scores. In addition, participants were examined only once, and controls were not assessed with the entire neuropsychological protocol, which would be interesting in order to evaluate test re-test reliability and differences between MS and healthy controls in associations between LASSI-L and other cognitive tests.

In conclusion, our study found a delay in learning and information acquisition, a difficulty in recovery after proactive semantic interference and, even more importantly, a retroactive semantic interference effect as the main characteristics in episodic verbal memory breakdowns in patients with MS. These findings are relevant in order to further understand memory impairment in MS, but also could be applicable in rehabilitation settings by minimizing both proactive and retroactive interference during early stages of memory consolidation. The LASSI-L showed good psychometric and diagnostic properties in MS, suggesting this instrument has utility for the neuropsychological assessment of MS patients. Due to our results about relevance of semantic interference, this study supports the use of memory tasks examining interference between word lists, which are generally omitted in cognitive batteries such as BICAMS using brief versions of the CVLT-II. Future studies with larger sample sizes and longitudinal designs are needed to evaluate potential clinical differences according to memory processes involved in subgroups of patients and the course of memory decline during the progression of MS according to LASSI-L. The correlation with structural and advanced neuroimaging tools may also provide new insights into the pathophysiology of memory deficits in MS. Furthermore, the comparison between LASSI-L performance and other widely used memory tests such as the CVLT which does not emphasize retroactive semantic interference or failure to recover from proactive semantic interference could help to further establish the utility of the LASSI-L for neuropsychological examination in the setting of MS.

## Data Availability Statement

The datasets generated for this study are available on request to the corresponding author.

## Ethics Statement

The studies involving human participants were reviewed and approved by Hospital Clinico San Carlos Ethics Research Committe. The patients/participants provided their written informed consent to participate in this study.

## Author Contributions

JAM-G: design of the study, data acquisition, statistical analysis, interpretation of data, writing of the manuscript, and final approval of the manuscript. AC-M: data acquisition, statistical analysis, literature review, and final approval of the manuscript. RC and DL: literature review, interpretation of data, critical revision of manuscript for important intellectual content, and final approval of the manuscript. AD-Á: data acquisition, design of the study, and final approval of the manuscript. AF-O: data acquisition, literature review, and final approval of the manuscript. VP: data acquisition, statistical analysis, and final approval of the manuscript. PM and TM-R: data acquisition, and study supervision. JM-G: study supervision, data acquisition, critical revision of manuscript for important intellectual content, and final approval of the manuscript.

## Conflict of Interest

The authors declare that the research was conducted in the absence of any commercial or financial relationships that could be construed as a potential conflict of interest.
