# Supplementary material for: Memory Impairment in Relapsing-Remitting Multiple Sclerosis Using a Challenging Semantic Interference Task
Source: Front Neurol. 2020 Apr 21;11:309. doi: 10.3389/fneur.2020.00309 (PMC7186484; doi:10.3389/fneur.2020.00309)
Supplement: Supplementary file 1 [file Table_1.DOCX]

**SUPPLEMENTARY MATERIAL**

| **Colors Legend** | <0.01 | No correlation |
| --- | --- | --- |
| \|0.01\| | \|0.199\| | Very Low correlation |
| \|0.2\| | \|0.399\| | Low correlation |
| \|0.4\| | \|0.699\| | Moderate correlation |
| \|0.7\| | \|0.899\| | High correlation |
| \|0.9\| | \|0.999\| | Very high correlation |

**S1. Correlations between LASSI-L scores and demographic factors.**

|  | FRA1 | CRA1 | CRA2 | FRB1 | CRB1 | CRB2 | SdFRA | SdCRA | DR |
| --- | --- | --- | --- | --- | --- | --- | --- | --- | --- |
| Age | -0.221* | -0.267* | -0.212* | -0.144 | -0.177 | -0.193 | -0.166 | -0.143 | -0.132 |
| Years of Education | -0.067 | 0.024 | -0.155 | -0.107 | -0.036 | -0.120 | -0.030 | -0.047 | -0.052 |
| EDSS | -0.029 | 0.05 | -0.103 | -0.007 | 0.138 | 0.003 | -0.061 | -0.049 | 0.012 |
| Years since diagnosis | -0.269** | -0.250** | -0.234* | -0.317** | -0.248** | -0.172 | -0.276** | -0.126 | -0.173 |

*: p-value <0.05 (two-sides)

**: p-value <0.01 (two-sides)

| **Table S2.** LASSI-L intrusions in HC and MS patients. Two-sample t test. | | | | |
| --- | --- | --- | --- | --- |
| Score | HC | MS | t-test | p-value |
| ti-FRA1 | 0.10±0.33 | 0.44±0.77 | -3.92 | <0.001 |
| ti-CRA1 | 0.28±0.64 | 0.72±0.99 | -3.70 | <0.001 |
| ti-CRA2 | 0.10±0.35 | 0.39±0.66 | -3.73 | <0.001 |
| ti-FRB1 | 0.40±0.84 | 0.85±1.12 | -3.21 | 0.002 |
| i-FRB1 | 0.40±0.78 | 0.85±1.16 | -3.25 | 0.001 |
| ti-CRB1 | 1.10±1.75 | 2.26±2.24 | -4.14 | <0.001 |
| i-CRB1 | 1.01±1.66 | 2.15±2.15 | -4.25 | <0.001 |
| ti-CRB2 | 0.74±1.26 | 1.63±1.71 | -4.23 | <0.001 |
| i-CRB2 | 0.71±1.12 | 1.49±1.59 | -4.04 | <0.001 |
| ti-SdFRA | 0.61±1.08 | 1.41±1.72 | -3.90 | <0.001 |
| i-SdFRA | 0.56±1.02 | 1.27±1.66 | -3.59 | <0.001 |
| ti-SdCRA | 1.11±1.44 | 2.28±2.10 | -4.59 | <0.001 |
| i-SdCRA | 1.06±1.42 | 2.00±1.91 | -3.99 | <0.001 |
| PIEr CRB1 | 0.10±0.15 | 0.21±0.19 | -4.54 | <0.001 |
| PIEr CRB2 | 0.05±0.08 | 0.11±0.11 | -4.31 | <0.001 |
| PIEr SdCRA | 0.10±0.13 | 0.19±0.17 | -4.31 | <0.001 |
| Statistically significant p-values are shown in bold (p<0.01). | | | | |
